# The Effect of the Gut Microbiota on Transplanted Kidney Function

**DOI:** 10.3390/ijms24021260

**Published:** 2023-01-09

**Authors:** Jarosław Przybyciński, Sylwester Drożdżal, Aleksandra Wilk, Violetta Dziedziejko, Kamila Szumilas, Andrzej Pawlik

**Affiliations:** 1 Department of Nephrology, Transplantology and Internal Medicine, Pomeranian Medical University, 70-111 Szczecin, Poland; 2 Department of Histology and Embryology, Pomeranian Medical University, 70-111 Szczecin, Poland; 3 Department of Biochemistry and Medical Chemistry, Pomeranian Medical University, 70-111 Szczecin, Poland; 4 Department of Physiology, Pomeranian Medical University in Szczecin, 70-111 Szczecin, Poland

**Keywords:** intestinal microflora, microbiota, renal graft, immunosuppressive treatment

## Abstract

The intestinal microflora is extremely important, not only in the processes of absorption, digestion and biosynthesis of vitamins, but also in shaping the immune and cognitive functions of the human body. Several studies demonstrate a correlation between microbiota composition and such events as graft rejection, kidney interstitial fibrosis, urinary tract infections, and diarrhoea or graft tolerance. Some of those changes might be directly linked with pathologies such as colonization with pathogenic bacterial strains. Gut microbiota composition also plays an important role in metabolic complications and viral infections after transplantation. From the other side, gut microbiota might induce graft tolerance by promotion of T and B regulatory cells. Graft tolerance induction is still an extremely important issue regarding transplantology and might allow the reduction or even avoidance of immunosuppressive treatment. Although there is a rising evidence of the pivotal role of gut microbiota in aspects of kidney transplantation there is still a lack of knowledge on the direct mechanisms of microbiota action. Furthermore, some of those negative effects could be reversed by probiotics of faecal microbiota trapoinsplantation. While diabetes and hypertension as well as BKV and CMV viremia are common and important complications of transplantation, both worsening the graft function and causing systemic injuries, it opens up potential clinical treatment options. As has been also suggested in the current review, some bacterial subsets exhibit protective properties. However, currently, there is a lack of evidence on pro- and prebiotic supplementation in kidney transplant patients. In the current review, we describe the effect of the microbiota on the transplanted kidney in renal transplant recipients.

## 1. Introduction

The ‘gut microbiota’ is the set of bacteria that colonize the gastrointestinal tract. It has evolved with the host over thousands of years, creating intricate and mutually beneficial relationships [1]. The literature estimates that the number of micro-organisms in the digestive tract exceeds 10^14^, which is about 10 times more bacterial cells than the number of human cells. Moreover, this number is more than 100 times greater in terms of genome content (microbiome) than the human genome. Due to the large number of bacterial cells in the host organism and the micro-organisms inhabiting it, it is often referred to as a ‘superorganism’ [2]. The intestinal microflora is the most numerous population of microbes in the human body, consisting of approximately 10 trillion cells. Its total weight ranges from 1.5–2 kg. The amount of bacteria in the gut gradually increases from 10^5^ in the jejunum to 10^12^ bacteria per gram of food content in the large intestine.

Renal recipients need to use immunosuppressive treatment to avoid graft rejection. However, the duration of proper function of a renal graft is still unsatisfactory. Over the years, this has been improved, but it is still too short. Immunosuppressive drugs affect the microbiota, including the gut microbiota, which probably has a negative effect on the renal graft. Since the duration of graft function is still too short, knowledge in the field of transplantology still needs to be expanded. In the current review, we describe the effect of the microbiota on the transplanted kidney in renal transplant recipients.

## 2. Gut Microbiota

It has been found that the large intestine is inhabited by four types of bacteria: Firmicutes, Bacteroidetes, Proteobacteria and Actinobacteria. Most of them are absolutely anaerobic bacteria, i.e., Bacteroides, Clostridium and Fusobacterium; however, we must remember that apart from such a large population of bacteria, the microflora also includes fungi of the genus *Candida* spp. [3]. The composition of the bacterial flora of the digestive tract reflects the physiological properties in a given part of the body [4]. The density and composition of the microflora are influenced by chemical, nutritional and immunological factors along the entire gut. The small intestine typically has high levels of acids, oxygen and antimicrobials, and has a short food transit time. These properties limit the growth of bacteria, so only fast-growing or anaerobic anaerobes with the ability to adhere to the intestinal epithelium will survive [5].

Contrary to the small intestine, conditions in the colon favour the development of a diverse community of bacteria, mainly anaerobes, which have the ability to use complex carbohydrates that are not digested in the small intestine. The colon has been shown to be dominated by Prevotellaceae, Lachnospiraceae and Rikenellaceae [6]. Several different important environmental factors are involved in the formation of the microflora. These include medical surgery, geography, depression, smoking and living conditions [7]. Xenobiotics, such as antibiotics, but not host-targeting drugs, shape the physiology and gene expression of the active human gut microbiome [8]. Treatment with antibiotics significantly disturbs both the short-term and long-term microbial balance. It reduces both the number and diversity of bacterial cultures. Antibiotics such as clarithromycin, clindamycin and metronidazole as well as ciprofloxacin have been shown to influence the structure of the microflora for different periods of time [9]. The exact effects and recovery time of the microflora after administration of an antibiotic seem to depend on the individual variability and the variability of the microflora before antibiotic therapy [9]. Recent studies in mice showed that the destruction of the microflora by antibiotics affected the secondary metabolism of bile acids and serotonin in the colon, causing delayed gastrointestinal motility [9]. Mice treated with antibiotics are also more susceptible to infection with antibiotic-related pathogens, *S. typhimurium* and *C. difficile*, due to the alteration of carbohydrate availability in the mucosa, which promotes their expansion into the gut [10].

**Figure 1:**
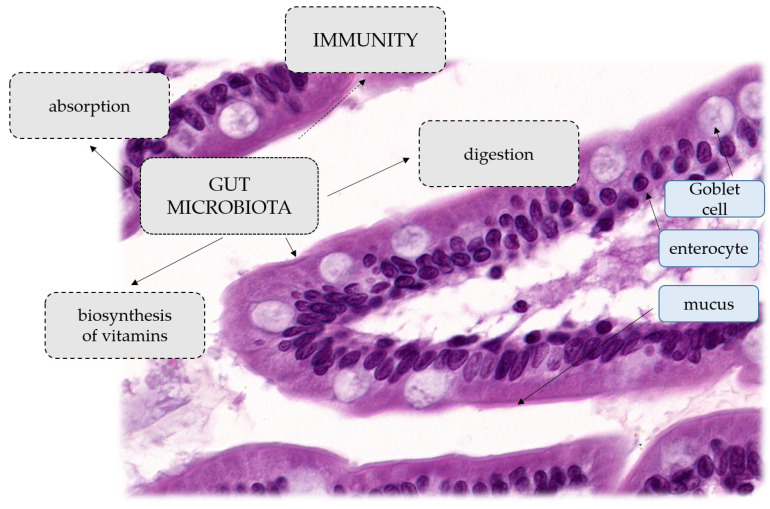
General functions of microbiota. Histological structure of the villus of the small intestine.

A human study has shown that the administration of intravenous β-lactam antibiotics consisting of ampicillin, sulbactam and cefazolin affects both microbial ecology and the production of key metabolites such as acetyl phosphate and acetyl-CoA, which are involved in major cellular functions [7]. Due to the high content of the genome, the intestinal microbiota provides the host with a number of beneficial properties. Some of the most important roles of these microbes are to help maintain the integrity of the mucus barrier, provide nutrients such as vitamins, or protect against pathogens. Moreover, the interaction between the commensal microbiota and the mucosal immune system is crucial for the proper functioning of the immune system. Bifidobacteria are the main producers of folic acid, a vitamin involved in important host metabolic processes including DNA synthesis and repair [11]. Lactic acid bacteria are key organisms in the production of vitamin B12, which neither animals, plants nor fungi can synthesise [12]. Other vitamins that the gut microbiota synthesises in humans include vitamin K, riboflavin, biotin, nicotinic acid, pantothenic acid, pyridoxine and thiamine [12]. In addition, several species of bacteria, such as *A. muciniphila* and *Lactobacillus plantarum*, are involved, as mentioned above, in promoting the integrity of the epithelium and also modulating the properties and transformation of mucus. It has been reported that mice kept under germ-free conditions have an extremely thin adherent layer of colon mucus, but when exposed to bacterial products (peptidoglycan or LPS), the thickness of the adherent mucus layer can be restored to levels seen in conventionally bred mice [13]. The gastrointestinal microbiota is also important for the development of the systemic immune system. The major immunodeficiency shown by germ-free animals is the lack of expansion of the CD4+ T cell population. This deficiency can be completely reversed by treating GF mice with polysaccharide A from the *B. fragilis* capsule [14]. This process is mainly mediated by pattern recognition receptors (PRRs) of epithelial cells, such as Toll-like or Nod-like receptors, which are able to recognise molecular effectors produced by gut microbes. These effectors mediate processes that can alleviate certain inflammatory bowel diseases, distinguish between beneficial and pathogenic bacteria, or increase the number of immune cells or PRRs [15]. The intestinal microflora is extremely important, not only in the processes of absorption, digestion and biosynthesis of vitamins, but also in shaping the immunity and cognitive functions of the human body. It also participates in the biotransformation of xenobiotics, and in the last few years it has been shown to influence the metabolism and pharmacokinetics of orally administered drugs, regulating their availability in the body [16]. General functions of microbiota and histological structure of the villus of the small intestine are presented on Figure 1.

## 3. Does the Microbiota Prolong Renal Graft Function?

Recent years have brought much needed attention to the role of the microbiota in renal transplantation. It is known that chronic kidney disease alters the gut microbiota, which has important clinical implications and might lead to severe complications. It is known that organ recipients need to use immunosuppressive drugs, and very often antibiotics that also affect the microbiota. Additionally, patients who have undergone organ transplantation have an altered appetite and bowel transit time. Furthermore, many common transplantation complications such as post-transplantation diabetes mellitus, diarrhoea, cardiovascular diseases, local and diffuse inflammation or infection have known associations with the gut microbiota. Moreover, the microbiota takes part in the metabolism of immunosuppressants and the tone of the human immunological system [17,18,19,20,21].

### 3.1. Changes in the Microbiota following Kidney Transplantation

Lee et al. analysed the gut microbiota from 26 patients before and early after kidney transplantation. They observed an increased abundance of Firmicutes with a decrease in Bacteroidetes. These were significant differences compared to healthy subjects. The relative abundance of Bacteroidetes decreased even more after transplantation and the phylum Proteobacteria increased. When comparing the gut microbiota of patients with or without diarrhoea, a reduction in Bacteroides, Ruminococcus, Coprococcus, and Dorea was observed in the first group. In addition, a study showed a reduction in Bacteroidetes with a reduction in Clostidiales and Bacteroidales in faecal samples during an episode of acute rejection. At the same time, there was an increase in Lactobacillales. Finally, the authors indicated a high abundance of Enterococcus in faecal samples from patients with an enterococcal UTI (urinary tract infection) [22]. Lee et al., in a further study, once again linked diarrhoea occurring after kidney transplantation with a lower Shannon index and dysbiosis in a cohort of 71 patients. They found decreased abundances of Eubacterium, Anaerostipes, Coprococcus, Romboutsia, Ruminococcus, Dorea, Faecalibacterium, Fusicatenibacter, Oscillibacter, Ruminiclostridium, Blautia, Bifidobacterium and Bacteroides and an increase in Enterococcus, Escherichia and Lachnoclostridium in patients with diarrhoea. The majority of those differences were independent from antibiotic use and time after transplantation. The authors did not identify the presence of common diarrhoea causing pathogens in all but two samples but found an increase in Ruminococcus abundance after the onset of diarrhoea. PICRUSt (Phylogenetic Investigation of Communities by Reconstruction of Unobserved States) analysis revealed lower metabolic function, starch and sucrose as well as amino and nucleotide sugar metabolism in the group with diarrhoea [22]. Swarte et al. compared the gut microbiota of kidney transplantation patients with healthy controls. They found lower microbiota diversity, as well as higher Proteobacteria and lower Actinobacteria abundances in transplant patients. Those changes were correlated to GFR (glomerular filtration rate) and PPI (proton pump inhibitor) and MMF (mycophenolate mofetil) use [23]. In a different study, Fricke et al. performed an analysis of the oral, rectal, urine and blood microbiota in 60 patients before and up to 6 months after kidney transplantation. The researched group had been treated with routine antibiotic prophylaxis with preoperative cefazoline or quinolone and prolonged sulfamethoxazole/trimethoprim as pneumocystis prophylaxis. They also received standard immunosuppression. They performed 16s RNA amplification to identify bacterial sequences. While they did not find bacteria in blood samples, urine showed bacterial abundance. In urine, 33% of samples were positive, of which 44% were dominated by a single bacterial genus. Those bacteria belonged to the genera *Lactobacillus, Enterococcus, Bifidobacteriaceae, Pseudomonas, Streptococcus* and *Corynebacterineae.* Rectal swabs obtained a microbiota consisting mainly of the phylum Firmicutes (more than 80% of bacterial phyla in 31 samples). Less common phyla included Bacteroides, Actinobacteria and Proteobacteria. Longitudinal analysis showed a reduction in the Shannon index after transplantation with the greatest microbiota shift occurring in samples before and 1 month after transplantation. Importantly, the authors were able to identify specific bacterial groups present before transplantation associated with later rejection episodes. They saw decreased *Leptotrichia, Neisseria,* and unknown members of Coriobacterineae and Corynebacterineae in oral swabs, and decreased *Anaerotruncus*, *Coprobacillus*, *Coprococcus* and an unknown member of the *Peptostreptococcaceae* in rectal swabs [24].

Rani et al. demonstrated alterations in the urinary microbiota after kidney transplantation. There was lower microbial diversity, an increase in Firmicutes (*Enterococcus faecalis*) and Proteobacteria (*Escherichia coli*) and a decrease in Actinobacteria in patients’ urine compared to healthy controls. The authors also showed an increase in folate metabolising enzymes and suggest an association with trimethoprim-sulfamethoxazole prophylaxis as a potential cause [25]. Modena et al. found a significant reduction in Streptococcus in urine in male patients after kidney transplantation compared with healthy controls. Moreover, this further declined in patients with biopsy-proven interstitial fibrosis/tubular atrophy [26].

**Figure 2:**
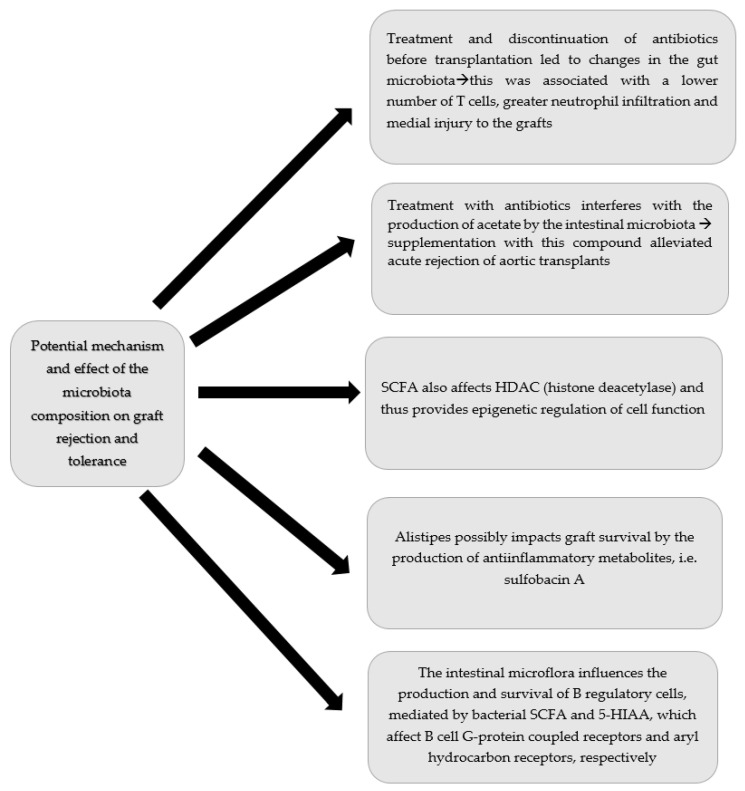
Potential influence of microflora on graft function.

The aforementioned studies revealed important changes in gut microbiota in patients after kidney transplantation. Interestingly there were significant differences among patients with or without common complications such as diarrhoea, and rejection of urinary tract infections. Some of those changes might be directly linked with pathologies such as colonization with pathogenic bacterial strains leading to urinary tract infections or diarrhoea. Others, such as rejection or some diarrhoea episodes could not be clearly explained by straightforward microbiota infection. Since the results are controversial, more researches regarding changes in the microbiota following kidney transplantation need to be performed. Potential influence of microflora on graft function is additionally presented at Figure 2.

### 3.2. Bidirectional Relationship between the Gut Microbiota and Immunosuppressive Treatment

Recent years have brought growing evidence that the gut microbiota plays a significant role in drug metabolism, transport and bioaccumulation, thereby affecting drug effectiveness and toxicity. On the other hand, some groups of drugs, such as antibiotics, PPI and NSAIDs, have been proven to significantly impact the animal and human microbiome [27]. In vitro studies suggest that up to 24% of human targeted drugs, among all drug classes, potentially inhibit bacterial growth [28]. On the contrary, it is also known that the tacrolimus concentration significantly increases during infectious diarrhoea, partially due to altered gut metabolism [29]. Tourret et al. showed the effects of immunosuppressive treatment on the gut microbiota in mice. They analysed faeces and ileal samples after 14 days of treatment with several types of immunosuppressant drugs, i.e., prednisolone, mycophenolate mofetil, tacrolimus, everolimus as well as combined therapy [30]. They found that prednisolone caused an increase in Firmicutes and a decrease in Bacteroidetes abundance in faeces. Furthermore, there was a significant decrease in *Clostridium sensu stricto* genus abundance in ileal samples in the prednisolone and combined therapy groups. Those changes might be partially explained by a decrease in IL-22 and C-type lectin secretion in the gut of treated mice. Interestingly, this effect was not observed in mice treated only with mycophenolate mofetil. There was also an increased risk of pathogenic *Escherichia coli* strain colonisation in treated mice. This shows important alterations in gut immunity during immunosuppression [30]. Mycophenolate mofetil treatment increases alpha diversity, the Firmicutes/Bacteroidetes ratio and the Shannon index in mice with inducted uveitis. There was also a decrease in Lachnospiraceae UCG-001, while Lachnospiraceae NK4A136 abundance increased. This in turn might be associated with Treg expansion. Interestingly, mycophenolate mofetil affected both the gut microbiota and the T cell population differently than methotrexate [31].

Zaza and colleagues analysed the gut microbial metagenetic profile of 20 stable kidney transplants recipients, depending on the type of maintenance immunosuppressive treatment. While all patients received mycophenolate mofetil 1000 mg b.i.d., nine patients additionally received everolimus and 11 received tacrolimus. Although the general composition of gut microbiota among both groups was similar with more than 50% of bacteria in the phylum Firmicutes (*Iuminococcaceae*, Lachnospiraceae, Streptococcaceae, Eubacteriaceae), differences in less abundant 11 OTU have been found. One of them was enriched in the tacrolimus plus mycophenolate mofetil group–*Haemophilus parainfluenzae* is considered an opportunistic pathogen. Furthermore, gene analysis showed significant enrichment in macrolide transport system msrA (*msrA)* in the regimen including everolimus plus mycophenolate mofetil. Flagellar motor switch protein *(fliNY)* and type IV pilus assembly protein pilM *(pilM)* were increased in tacrolimus plus mycophenolate mofetil. Those genes might potentially affect bacterial virulence. Besides immunosuppressive treatment, the study showed that the gut microbiota is affected by the consumption of sugar [32].

Zhang et al. demonstrated that tacrolimus treatment changes the microbiota structure in mice. In this study, mice in the tacrolimus group showed a higher abundance of *Allobaculum*, *Bacteroides* and *Lactobacillus.* On the other hand, there was a decrease in *Clostridium, Ruminococcus, Rikenella, Ruminococcaceae* and *Oscillospira* abundance on day 14 of treatment. Those alterations caused significant changes in microbiota function, including SCFAs (short chain fatty acids) metabolism, based on PICRUSt analysis based on the 16s RNA composition. Moreover, tacrolimus treatment as well as faecal microbiota transplantation from mice treated with high dose of tacrolimus, increased the Treg population in blood and mesenteric lymph nodes. Finally, FMT from mice treated with a high dose of tacrolimus, or with a low dose of tacrolimus, significantly improved skin graft survival in mice [33].

Lee and colleagues performed an analysis of the gut microbiota using deep sequencing of the PCR amplified 16S rRNA V4-V5 region in a group of 19 patients one month after kidney transplantation. In this study, they showed an association of *Feacalibacterium prausitzii* abundance and tacrolimus dosing requirements. In the group needing escalation of tacrolimus dosing to maintain the target drug concentration, *Feacalibacterium prausitzii* represented 11.8% of the gut microbiota compared with 0.8% in the drug stable group one week after transplantation [34]. Similarly, Jennings et al. found a relationship between gut microbiota diversity and tacrolimus dosing requirements in patients early after heart transplantation [35]. On the other hand, Woodworth et al. reviewed 10 cases of solid organ transplantation patients and found non-significant differences in tacrolimus dosing after FMT for treatment of CDI. Recently, Guo and colleagues identified that *Feacalibacterium prausitzii* and other Clostridiales are capable of metabolising tacrolimus to less active metabolites. In an in vitro study, they showed that *Feacalibacterium prausitzii* and other Clostridiales might turn tacrolimus into an C9-keto-reduction product. Further studies revealed that this metabolite is approximately 15-fold less potent as an immunosuppressant and antifungal drug. Other bacteria capable of performing the same in vitro reaction are Erysipelotrichales and to a small extent Bacteroidales. Bifidobacteriales failed to produce this metabolite [36]. Recently, Quian and colleagues identified new enzymes, widely present among Clostridioides, involved in tacrolimus metabolism [37].

Other data pointing out the importance of the microbiota in tacrolimus metabolism is the change in its trough concentration after antibiotic treatment that cannot be attributed to direct drug–drug relationships. Zheng et al. analysed the tacrolimus trough level and concentration over a dose ratio change in kidney transplant patients before and after antibiotic therapy. They found that cephalosporins and penicillin-type antibiotics significantly altered those values while fluoroquinolones did not have a significant effect [38].

Mycophenolate mofetil is an immunosuppressive drug currently considered as a staple of post-transplantation treatment. Its active metabolite is mycophenolic acid, which is afterwards deactivated by glucuronidation and undergoes enterohepatic recirculation. The degree of this process impacts not only drug function but also possible side-effects such as diarrhoea. Simpson and colleagues examined faecal samples of patients after kidney transplantation and identified differences in bacterial β-glucuronidase function compared to healthy individuals. They were able to find a higher abundance of a flavin mononucleotide-binding ortholog of β-glucuronidase, causing reactivation of mycophenolic acid [39]. Moreover, Khan et al. revealed that there is a difference in GUS (β-glucuronidase) activity in stool between hematopoietic cell and kidney transplantation patients, possibly affecting mycophenolate mofetil dosing requirements. In this study, kidney transplantation patients had greater GUS activity, which might be caused by different treatments (chemotherapy, antibiotics) preceding hematopoietic cell transplantation [40]. It has been previously observed that amoxicillin/clavulanate reduces the MPA (mycophenolic acid) plasma concentration in rats with preserved enterohepatic circulation [41]. Flanigan and colleagues demonstrated that gastrointestinal toxicity, colonic inflammation and weight lost in mice treated with mycophenolate mofetil are associated with changes in the gut microbiota. Loss of bacterial diversity with a higher abundance of Proteobacteria such as *Escherichia* and *Shigella* strains has been observed. Interestingly, this effect was not detected in germ-free mice or mice treated with broad-spectrum antibiotics. Furthermore, colonic inflammation could be ameliorated with antibiotic administration after 8 days of mycophenolate mofetil treatment. Further studies on mice revealed that gastrointestinal tract toxicity might be linked with direct MPA exposure after bacterial deglucuronidation. There was a change in the gut microbiota composition after mycophenolate mofetil treatment with the abundances of *Betaproteobacteria, Bacteroidia, Bacilli, Gammaproteobacteria, Erysipelotrichia*, and *Alphaproteobacteria* related to increased activity of GUS in the mouse proximal colon. Interestingly, these effects were mitigated with co-administration of vancomycin, but not metronidazole, after 8 days of mycophenolate mofetil treatment. It caused *Bacteroidia, Clostridia* and *Erysipelotrichia* taxa depletion, a reduction in GUS activity and reduced colon inflammation [42]. In line with this research, Saqr et al. found that higher blood MPA concentrations in patients after intravenous mycophenolate mofetil treatment in the course of hematopoietic cell transplantation was related to enterohepatic drug recirculation and the gut microbiota. In this study, patients with higher blood MPA concentrations had a greater abundance of *Bacteroides vulgatus*, *Bacteroides stercoris* and *Bacteroides thetaiotaomicron* compared with patients with lower enterohepatic recirculation. There was a negative correlation between enterohepatic recirculation with *Blautia hydrogenotrophica* genus abundance [43]. Another similar study demonstrated reduced gut microbiota diversity in patients with diarrhoea. Furthermore, faecal β-glucuronidase activity was positively correlated with diarrhoea duration and could be a marker of mycophenolate mofetil toxicity [33]. Jardou et al. revealed that mycophenolate mofetil treatment reduces the SCFA concentration in faeces and blood in mice. In this study, there was a significant reduction in faecal acetate and propionate and serum butyrate after 8 days of mycophenolate mofetil [44].

On the other hand, Robles-Vera et al. linked mycophenolate mofetil treatment with a reduction in dysbiosis, improvement of gut integrity and reduction in neuroinflammation causing the sympathetic gut drive in hypertensive rats. In this study, mycophenolate mofetil treatment reduced Firmicutes and *Lactobcillus* abundance while increasing Bacteroidetes and *Sutterella*, making the microbiota composition similar to normotensive rats. Generally, mycophenolate mofetil treatment increased the abundance of SCFA-producing bacteria which then affected the gut immune system via Treg cells [45]. Additionally, there is growing evidence of a vital role of mTOR signalling in innate antibacterial immunity and autophagy of infected cells and bacteria, but there is a lack of studies regarding the effect of mTOR inhibition on the human gut microbiota [46].

Glucocorticoids also significantly modify the gut microbiota [18,47]. Tao et al. showed a reduction in the Shannon index of the gut microbiota in rats and modification of their circadian rhythm [48]. Interestingly, the therapeutic effect of prednisone treatment might be augmented by microbiota alterations. He et al. demonstrated decreases in Mucispirillum, Oscillospira, Bilophila and Rikenella, and an increase in Anaerostipes after prednisone administration in a mouse lupus model. Further alterations to the gut microbiota with bromofuranone caused an increase in *Lactobacillus, Allobaculum, Suterella* and *Adlercreutzia* and potentiated the action of prednisone [49]. In addition, the gut microbiota can metabolise glucocorticoids to androgens, changing their immunosuppressive potential [50]. There are far fewer studies regarding the effect of other immunosuppressive agents on the gut microbiota. Cyclosporin did not affect the human gut microbiota in in vivo and ex vivo studies [51]. In a different study, it led to the restoration of microbiota structure in rats with acute liver rejection. Azathioprine was capable of inhibiting the growth of some bacteria such as *Clostridium consisus*, *Escherichia coli* and *Bacteroides fragilis* and *vulgatus* in an in vitro study [52]. Li et al. demonstrated in a monkey model that microbiota alterations are associated with gut lymphocyte depletion after alemtuzumab [53].

Aforementioned studies showed the important role of gut microbiota on immunosuppressive drug metabolism. The best-known example is *Feacalibacterium prausitzii* changing the metabolism of tacrolimus. It might lead to unexpected shifts in tacrolimus concentration, causing either drug toxicity or rejection episodes. On the other hand, other bacteria might increase MMF toxicity by increasing gut MPA exposition. Most common is when diarrhoea causes dehydration and, in turn, graft injury. Furthermore, in most cases, lowering the MMF dose can increase potential rejection episodes. Besides interaction with immunosuppressive drugs, gut microbiota might affect a transplanted kidney through various other mechanisms. For example, gut microbiota interact with immune cells and endothelium both directly and through metabolites such as SCFAs, tryptophan derivates, bile acids and amines [54,55]. Several studies demonstrated correlation between microbiota composition and such events as graft rejection, kidney interstitial fibrosis, urinary tract infections, diarrhoea or graft tolerance. Although there is a rising evidence of the pivotal role of gut microbiota in the course of kidney transplantation, there is still lack of knowledge over direct mechanisms of microbiota action [21].

### 3.3. Relationship between the Gut Microbiota and Transplantation Complications

New onset of diabetes mellitus after transplantation is a common complication with a complex pathophysiology and increased mortality burden. There is increasing evidence linking impaired glucose metabolism with the gut microbiota composition [56]. Jiao et al. analyzed the effect of tacrolimus treatment on the mouse microbiota and the incidence of post-transplantation diabetes mellitus. They found that tacrolimus significantly changed the composition of the gut microbiota, most notably by increasing *Alistipes*, *Allobaculum* and *Bacteroides* while decreasing *Lachnospiraceae_NK4A136, Akkermansia* and *Ruminococcaceae.* Furthermore, mice treated with tacrolimus had lower butyric and isovaleric acid concentrations in the cecum faeces. Moreover, tacrolimus treatment caused elevated fasting blood glucose, HbA1c and OGTT (oral glucose tolerance) test results. This effect was mitigated in mice receiving antibiotics and tacrolimus simultaneously. Additionally, the tacrolimus-mediated diabetogenic effect could be reduced by oral butyrate supplementation. In this study, butyrate restored the excretion of various hormones, GLP-1 (Glucagon-Like Peptide-1), PYY (peptide YY) and insulin [57]. Bhat et al. observed a decrease in *Roseburia, Oscillospira, Mollicutes, Rothia, Micrococcaceae, Actinomycetales* and *Staphylococcus* abundances in hyperglycaemic rats treated with tacrolimus or sirolimus. There was an increase in the *Lactobacillus* genus and Akkermansia muciniphila. Further analysis of metabolic pathways involved in specific bacteria revealed a decrease of starch degradation and butyrate production. On the other hand, there was an increase in catabolism and sucrose degradation caused by Lactobacillus johnsonii and Lactobacillus ASF360, a phenomenon earlier observed in diabetes. Moreover, hyperglycaemia could be ameliorated using probiotic treatment with Lactobacillus plantarum spp [58]. Han et al. showed a cumulative negative effect on the gut microbiota, metabolic profile and diabetes in mice treated with tacrolimus and antibiotics. They found a significant reduction in the Firmicutes genus *Coprococcus* in this group. In humans, Lecronier et al. showed a significant increase in *Lactobacillus* spp. and a decrease in Akkermansia muciniphila in patients with NODAT (new onset diabetes after transplantation) or pre-existing diabetes compared with non-diabetic patients [59].

Another common tacrolimus adverse effect is blood hypertension. Toral et al. demonstrated that it might be mediated by gut dysbiosis. In this study, mice treated with tacrolimus had lower microbiota diversity, increased Firmicutes/Bacteroidetes and lower SCFA production. In parallel, they had higher vascular oxidative stress and an altered Th17/Treg mesenteric balance. Vascular abnormalities could be partially reversed by *Lactobacillus fermentum* CECT5716 supplementation of faecal microbiota transplantation from mice receiving these bacteria [60].

Interestingly, Lee et al. found a correlation between the abundance of butyric acid producing bacterial and viral infections in patients after kidney transplantation. In this study, a relative abundance of butyric acid producing bacteria < 1% of the gut microbiota increased the risk of infection without association with BKV (BK virus) and CMV (cytomegalovirus) viremia [22].

These studies show that gut microbiota composition plays an important role in metabolic complications and viral infections after transplantation. The authors explain this by different sucrose metabolism and SCFAs secretion by gut microbiota. Furthermore, some of those negative effects could be reversed by probiotics of faecal microbiota transplantation. While diabetes and hypertension as well as BKV and CMV viremia are common and important complications of transplantation, both worsening the graft function and causing systemic injuries, it opens potential clinical treatment options.

### 3.4. Effect of the Microbiota Composition on Graft Rejection and Tolerance

Despite advances in the field of immunosuppressive treatment, graft rejection remains one of the major complications. It is often initiated by altered metabolism and a high concentration of immunosuppression or loss of immunological balance caused by an infection, bringing attention to the role of the gut microbiota in this process. In fact, bacterial-colonised allografts such as skin might be rejected earlier partially due to host vs. commensal immune responses [61]. Rey et al. showed that microbiota alterations through an antibiotic cocktail in early mouse life exacerbated acute vascular rejection of aortic grafts. In this model, even three weeks of treatment and discontinuation of antibiotics before transplantation led to persistent changes in the gut microbiota, with a reduction in Bacteroidia and a slight increase in Clostridia. This was associated with a lower number of Treg cells, greater neutrophil infiltration and medial injury to the grafts [62]. The same group demonstrated later that antibiotic treatment in early life in female mice impaired acetate production by the gut microbiota. Normalisation of the microbiota by co-housing mitigated the acute rejection of aortic grafts. A similar, positive effect could be achieved by acetate supplementation [62]. Wang et al. were able to characterise significant differences in the gut microbiota composition of patients with antibody-mediated rejection of kidney allografts. Specific taxa, i.e., *Lactobacillales, Erysipelotrichi, Erysipelotrichales, Erysipelotrichaceae, Bacilli, Clostridia, Clostridiales* and *Roseburia* showed different abundances. The most significant alteration was the reduction in *Clostridia* and *Clostridiales* in the antibody-mediated rejection group, which might function as a marker of this process. The authors also revealed differences in microbiota-associated functional pathways in PICRUSt analysis, but it is still too early to show a direct role of the microbiota in AMR (antimicrobial resistance) [63].

Another important aspect is the effect of the gut microbiota on allograft tolerance. It is well known that bacterial SCFAs can promote Treg differentiation [64]. McIntosh et al. were able to demonstrate the impact of different microbiota compositions on skin graft rejection in mice. They compared populations of mice with the same genetic background acquired from different vendors, thus having different microbiota. They found that higher abundance of the *Alistipes* genus delayed skin graft rejection. Furthermore, they were able to project this tolerogenic effect on different mice groups by faecal microbiota transplantation or cohousing. *Alistipes* possibly impacts graft survival by the production of anti-inflammatory metabolites, i.e., sulfobacin A [65]. There is increasing evidence pointing out a role for Breg cells in tolerogenic environment formation. It has been shown that the gut microbiota, for example *Clostridia,* affects B regulatory cell generation and survival, mediated through bacterial SCFAs and 5-HIAA which affect B cell G-protein coupled receptors and aryl hydrocarbon receptors, respectively. SCFA also affects HDAC (histone deacetylase) and thus provides epigenetic regulation of cell function, which seems to be dose-dependent [66]. Similarly, Alhababb et al. revealed significant differences in skin graft survival among groups of mice after B cell transfer obtained from sterile or conventional environment housed animals. Mice housed in a non-sterile environment had decreased abundance of *Lachnospiraceae* and *Bifidobacterium* groups and had more potent regulatory B cells. This effect was not seen in mice that had been treated with antibiotics prior to B cell transfer [67]. On the other hand, another study showed that microbiota alterations caused by antibiotic pretreatment promote skin graft survival in a murine model [68]. Wu et al. pointed out that a high-fibre diet could protect against dysbiosis after kidney allograft transplantation in mice without immunosuppressive treatment. High-fibre diet mice had a higher abundance of *Bifidobacterium* spp., *Bacteroides* spp. and *Clostridiales* sp. which can produce SCFAs. Furthermore, high-fibre mice showed better kidney graft function at days 14 and 100. They presented lower serum creatinine concentrations and less profound histological lesions in the graft. Interestingly, a similar protective effect could be achieved by SCFA supplementation using sodium acetate. In this study, a high-fibre diet caused a higher abundance of SCFA-producing bacteria and SCFA-promoted Treg cell development through the GPR43 receptor [69,70].

Colas et al. analyzed the urinary microbiome of different populations of patients after kidney transplantation. They identified a relative increase of abundance and a distinct profile of the Proteobacteria phylum in spontaneously tolerant patients (with stable graft function without immunosuppressive treatment). There was an increase in Oxalobacteraceae (genus *Janthinobacterium)*, Caulobacteraceae, Comamonadaceae, Moraxellaceae (genus *Acinetobacter),* Xanthomonadaceae*, Achromobacter* and *Yersinia* abundance. Immunosuppressive treatment also impacted the urine microbiota. CNI (calcineurin inhibitor) and mTOR inhibitors decreased *Lactobacilales* while glucocorticoids increased *Clostridia.* In this study, the distinct microbiota profile was sex dependent and stable over time [71].

Notably, Kim et al. revealed that differences between the gut microbiota of living donors and recipients of kidney allograft might impact transplantation outcomes [72].

Importantly, new research has shown that the gut microbiota can induce transplant tolerance by promoting T and B regulatory lymphocytes. Induction of graft tolerance may allow for reduction or even withdrawal of immunosuppressive treatment. The data seem very promising, but need to be expanded.

### 3.5. Role of the Microbiota Metabolites after Kidney Transplantation

It is well known that uremic toxins contribute to kidney injury and CKD complications. Many uremic toxins are gut microbiota co-metabolites. Among them, indoxyl sulfate and *p*-cresyl sulfate are end products of protein fermentation, while trimethylamine-*N*-oxide originates from carnitine and choline metabolism [73]. In CKD, those toxins accumulate together with GFR decline and might contribute to endothelial dysfunction, inflammation and oxidative stress. Their action leads to cardiovascular events, mortality as well as cerebrovascular and cognitive disorders in humans and animal models [74,75]. Recently, Poesen et al. demonstrated a significant decrease in several toxic co-metabolites, but not trimethylamine-N-oxide (TMAO) after kidney transplantation. Interestingly patients after kidney transplantation also had a lower co-metabolite urine excretion rate showing complex and a not yet fully understandable mechanism. It is speculated that changes in microbiota composition, absorption, transport and metabolism by human enzymes, not only GFR increase, might be responsible for those results [76]. Similarly, Liabeuf et al. showed a rapid decline of plasma indoxyl sulfate in patients after kidney transplantation. This study showed a lack of correlation between plasma indoxyl sulfate and mortality or cardiovascular complications in a 12-month observation [77]. In the following study, Liabeuf et al. showed decline in plasma indole acetic acid after kidney transplantation. Moreover, IAA plasma concentration in transplanted patients was lower compared to healthy control with matched GFR. While IAA was a marker of increased mortality and cardiovascular complication risk in patients with CKD, it did not show this correlation after kidney transplantation [77]. Kouidhi et al. revealed significant differences in faecal metabolites composition between stable kidney graft recipients and healthy control. Notably, there was a decline in faecal SCFAs concentrations in patients after transplantation. Faecal metabolomics could provide useful information and clinical application but need further study [78]. Overall, there is still little knowledge regarding the role of microbiota-derived co-metabolites in graft function and complications after kidney transplantation.

## 4. Conclusions

To sum up, there is still little knowledge regarding the role of microbiota-derived co-metabolites in graft function and complications after kidney transplantation. However, several studies demonstrated a correlation between microbiota composition and such events as graft rejection, kidney interstitial fibrosis, urinary tract infections, diarrhoea or graft tolerance. Some of those changes might be directly linked with pathologies such as colonization with pathogenic bacterial strains. Gut microbiota composition also plays an important role in metabolic complications and viral infections after transplantation.

Of note, gut microbiota might induce graft tolerance by promotion of T and B regulatory cells. Graft tolerance induction might allow a reduction or even avoidance of immunosuppressive treatment. Although there is rising evidence of the pivotal role of gut microbiota in aspects of kidney transplantation there is still a lack of knowledge over direct mechanisms of microbiota action.

Furthermore, some of those negative effects could be reversed by probiotics of faecal microbiota transplantation. While diabetes and hypertension as well as BKV and CMV viremia are common and important complications of transplantation, both worsening the graft function and causing systemic injuries, it opens potential clinical treatment options. As it has been also suggested in the current review, some bacterial subsets exhibit protective properties. However, currently, there is a lack of evidence on pro- and prebiotic supplementation in kidney transplant patients.

## Data Availability

Not applicable.
